# RCT on the effectiveness of the intraligamentary anesthesia and inferior alveolar nerve block on pain during dental treatment

**DOI:** 10.1007/s00784-021-03787-x

**Published:** 2021-02-01

**Authors:** Bahaa R. Youssef, Andreas Söhnel, Alexander Welk, Mohamed H. Abudrya, Mohamed Baider, Mohammad Alkilzy, Christian Splieth

**Affiliations:** 1 Departments of Preventive Dentistry and Pediatric Dentistry, University Medicine of Greifswald, Fleischmannstr. 42, 17487 Greifswald, Germany; 2 Department of Prosthetic Dentistry, Gerodontology and Biomaterials, University Medicine of Greifswald, Greifswald, Germany; 3 Department of Restorative Dentistry, Periodontology and Endodotontology, University Medicine of Greifswald, Greifswald, Germany

**Keywords:** Local anesthetic, Dentistry, Pain, Inferior alveolar nerve block, RCT

## Abstract

**Objective:**

To compare the effectiveness and complications of intraligamentary anesthesia (ILA) with conventional inferior alveolar nerve block (IANB) during injection and dental treatment of mandibular posterior teeth.

**Materials and methods:**

In this randomized, prospective clinical trial, 72 patients (39 males, 33 females), scheduled for dental treatment of mandibular posterior teeth, were randomly allocated to ILA group (*n* = 35) received ILA injection or IANB group (*n* = 37) received the conventional IANB. Our primary outcome was to assess pain and stress (discomfort) during the injection and dental treatment, using the numeric rating scale (NRS) from 0 to 10 (0 = no pain, 10= the worst pain imaginable), whereas recording 24-h postoperative complications was our secondary outcomes.

**Results:**

Patients in ILA group reported significantly less pain during injection when compared with IANB group (*p* = 0.03), while pain during dental treatment was similar in both groups (*p* = 0.2). Patients in both groups also reported similar law values of discomfort during treatment (*p* = 0.7). Although no signs of nerve contact or any other postoperative complications were observed, five patients in IANB group (none in ILA group) reported temporary irritations.

**Conclusion:**

This study showed equivalent effectiveness of both intraligamentary anesthesia and conventional inferior alveolar nerve block, for pain control during routine dental treatment of mandibular posterior teeth. Nevertheless, ILA showed significantly less pain during injection. No major postoperative complications in both groups were observed.

**Clinical relevance:**

ILA could be considered as an effective alternative for routine dental treatment.

**Trial registration:**

NCT04563351

**Supplementary Information:**

The online version contains supplementary material available at 10.1007/s00784-021-03787-x.

## Introduction

Pain is a relevant problem in dental treatment [1], making the administration of local anesthetics a necessary and routine measure for various dental procedures [2]. Unfortunately, pain, side effects, and a widely common fear of the injection are also relevant issues in dentistry [3], often resulting in missed or delayed appointments [4]. Regarding local anesthesia for mandibular teeth, two alternative techniques are well established: inferior alveolar nerve block (IANB) or local intraligamentary anesthesia (ILA) at the treated tooth. For decades, IANB has been considered as gold standard for blocking the hemimandible [5], especially in posterior mandibular permanent teeth. It provides adequate anesthesia for one side of the mandibular teeth and gingival mucosa, the body and inferior ramus of the mandible, and the anterior two-thirds of the tongue and floor of the mouth effectively [5, 6].

The evidence suggests that IANB is relatively painful and has a comparatively higher failure rate [7]. It also has a technique-immanent risk, such as transient or even persistent damage to the lingual and/or the inferior alveolar nerve [8]. Moreover, it may provoke intravascular injections, hematoma, muscle injury, and trismus [9]. In addition, IANB is associated with an increased risk of burning sensations and/or bite injuries, especially in children and patients with mental disorder due to the long duration of the soft tissue anesthesia which exceeds the dental treatment time considerably. In consequence, an alternative, local, and tooth-based anesthetic technique is demanded [9], which ILA could present.

A considerable number of literatures on ILA as alternative technique for IANB were generated over the last years [10, 11]. ILA only requires an injection directly into the periodontal space of the tooth with relatively high pressure. The injected solution spreads to the cancellous bone adjacent to the tooth to be anesthetized [12, 13]. Among the advantages of this technique are the rapid onset of action, a reasonable duration of 30–49 min, which is in line with standard dental treatment, as well as a low and safe amount of anesthetic solution (about 0.2 ml for each root) [10]. It is of high safety in pediatric patients, patients with bleeding disorders as well as in medically compromised patients [9, 14, 15]. On the other hand, ILA has its own limitations, especially because it is applied for single teeth and bacteremia is reported [10]. Also, the question of (reversible) damage of periodontal tissue, bone, and even root resorption is discussed [10]. In some procedures such as extractions, this is not relevant at all. In summary, current research views ILA as possible good alternative to IANB for dental routine procedures [10, 11]. Therefore, the aim of this study was to evaluate the effectiveness of ILA versus IANB for dental treatment of mandibular posterior teeth.

## Materials and methods

This prospective randomized comparative clinical trial was conducted in the integrated clinical course in the dental school of the University of Greifswald, Germany, after the approval of the local ethics committee of the medical faculty in Greifswald (No. BB 174/18) in a period from December 2018 to June 2019. It was also registered in ClinicalTrails.gov (ID: NCT04563351). The sample size calculation using “G*power version 3.1” (Heinrich-Heine-University/Germany) was based on the following estimates: *T* test for means (difference between two independent means), effect size 0.7, *α* error 0.05, and power (1-ß error) 0.9. It resulted in a samples size of 36 patients in each of the two groups (IANB and ILA).

### Inclusion and exclusion criteria

The patients requiring regular dental treatment in permanent mandibular posterior teeth under local anesthesia were recruited with an age range of 18 to 50 years. Patients were not included if they had a clinical or radiographic sign of acute abscess, pus, or peri-radicular pathology. Also, patients with a systemic disease requiring special considerations during their dental treatment or patients with contra-indications for any of the components of the anesthetic solution (allergy to articaine, epinephrine, and sulfite) were excluded.

### Clinical treatment and outcome

**Fig. 1:**
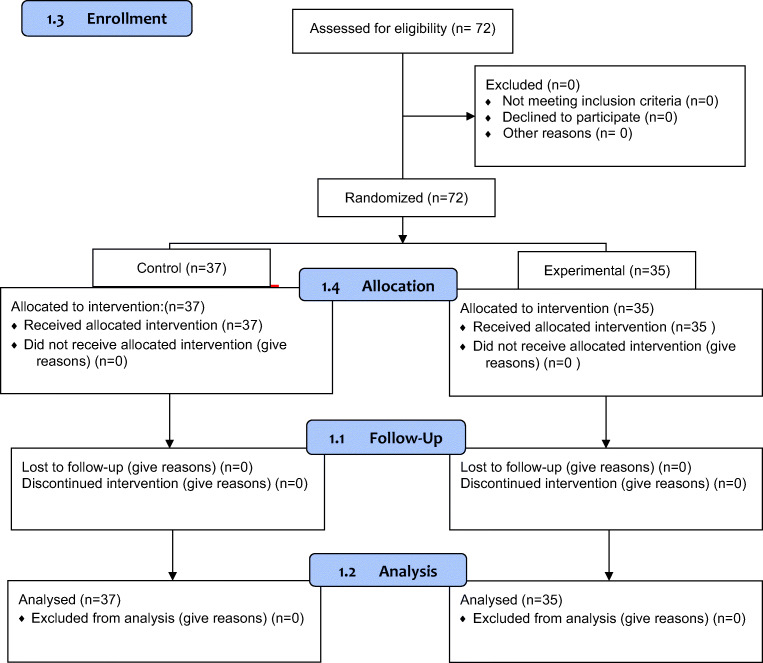
Show the CONSORT diagram

Only one tooth per patient was included in the study. A computer-generated random number list with allocation concealment was used to assign patients to one of the two groups (ILA vs. IANB, see CONSORT diagram, Fig. 1). The dental practitioners (GDPs) or the students performing the treatment and evaluating the effectiveness of the anesthesia were blinded to the form of anesthesia which was administered by the clinical instructors of the course being also dental practitioners (GDPs) or other dental students in the 4th and 5th academic year in the integrated clinical course in the dental school of the University of Greifswald. The distributions of different experience level of clinical instructors, dental students in the 4th and 5th year were recorded and analyzed. The intensity of pain as well as stress during the injection of the local anesthesia and during the dental procedure was assessed by using the numeric rating scale (NRS 0–10). For the inferior alveolar nerve block, the patient was placed comfortably in a supine position on the dental chair. The start of the anesthetic procedure was done without using topical anesthesia. The IANB injection was administered with cannulas of 38 mm in length and a gauge of 0.4 mm (Sopira Carpule, Heraeus Kulzer GmbH Hanau, Germany). The patients were anesthetized with Ultracain DS Forte 1:100.00 (Sanofi-Aventis, Germany), the active ingredient being articaine in 1.7-ml ampules (1 ml equal to 40 mg articaine hydrochloride and 0.012 mg epinephrine hydrochloride, which is included as a vasoconstrictor). Once the bone was contacted, 1.5 ml of anesthetic solution was injected slowly [16, 17]. Subsequently, the needle was detached for approximately 1 cm and an addition of 0.3–0.5 ml of local anesthetic solution was injected to anesthetize the lingual nerve [6].

For the intraligamentary anesthesia, three different syringe systems were used with randomized selection: Softjet syringe (Henke-Sass Wolf, Tuttlingen, Germany), Citojet syringe (Sopira, Heraeus Kulzer GmbH Hanau, Germany), and Ultrajet syringe (Sanofi-Aventis, Frankfurt am Main, Germany). The patients were also placed in a supine position and the dentist administered the ILA injection without using topical anesthesia with cannulas of 12 mm in length and a gauge of 0.30 mm (Sopira Carpule, Heraeus Kulzer GmbH Hanau, Germany). Also, Ultracain DS Forte 1:100.000 (Sanofi-Aventis, Germany) was used from 1.7-ml ampules. The needle was navigated through the gingival sulcus with the bevel towards the alveolar bone and away from the root surface, at an angle of 30°–40° to the long axis of the tooth and 2–3 mm into the periodontal ligament space between root and alveolar bone. For each root, 0.2 ml of local anesthetic was injected over at least 20 s according to Endo et al. (2008) as well as Bender and Taubenheim (2014) [15].

The intensity of pain during the injection of the LA and during the dental treatment was the primary outcome, assessed by the patient using the numeric rating scale (NRS) 0–10 (0 representing no pain at all and 10 representing the worst pain imaginable).

The secondary outcomes were postoperative complications, temporary irritation, and the duration of the anesthesia (when the feeling of numbness had disappeared) being assessed after 24 h by calling the patient.

### Statistical analyses

Skewedness, quartiles, and standard deviations were checked for the distribution of the data. For the descriptive analysis of categorical data, absolute and relative frequencies were calculated. For continuous data, minimum, maximum, median, and mean values. Categorical data were visualized via bar charts and consistent data via boxplots. For further explorative data analysis, Kolmogorov-Smirnov test was employed to test for a difference among ILA and IANB. In cases of *p* values < 0.05, Mann-Whitney *U* test and Kruskal-Wallis H, and in cases of *p* values > 0.05, Student’s *t* test and one-way ANOVA for independent samples were employed for the main analysis between ILA and IANB. In addition, the data for the different ILA syringe systems were compared in a sub analysis. The influence of categorical variables was shown with chi-square tests and cross tables. A significance level was set at 0.05. All analyses were carried out using SPSS Statistics version 22 (IBM, Armonk, NY, USA).

## Results

**Fig. 2:**
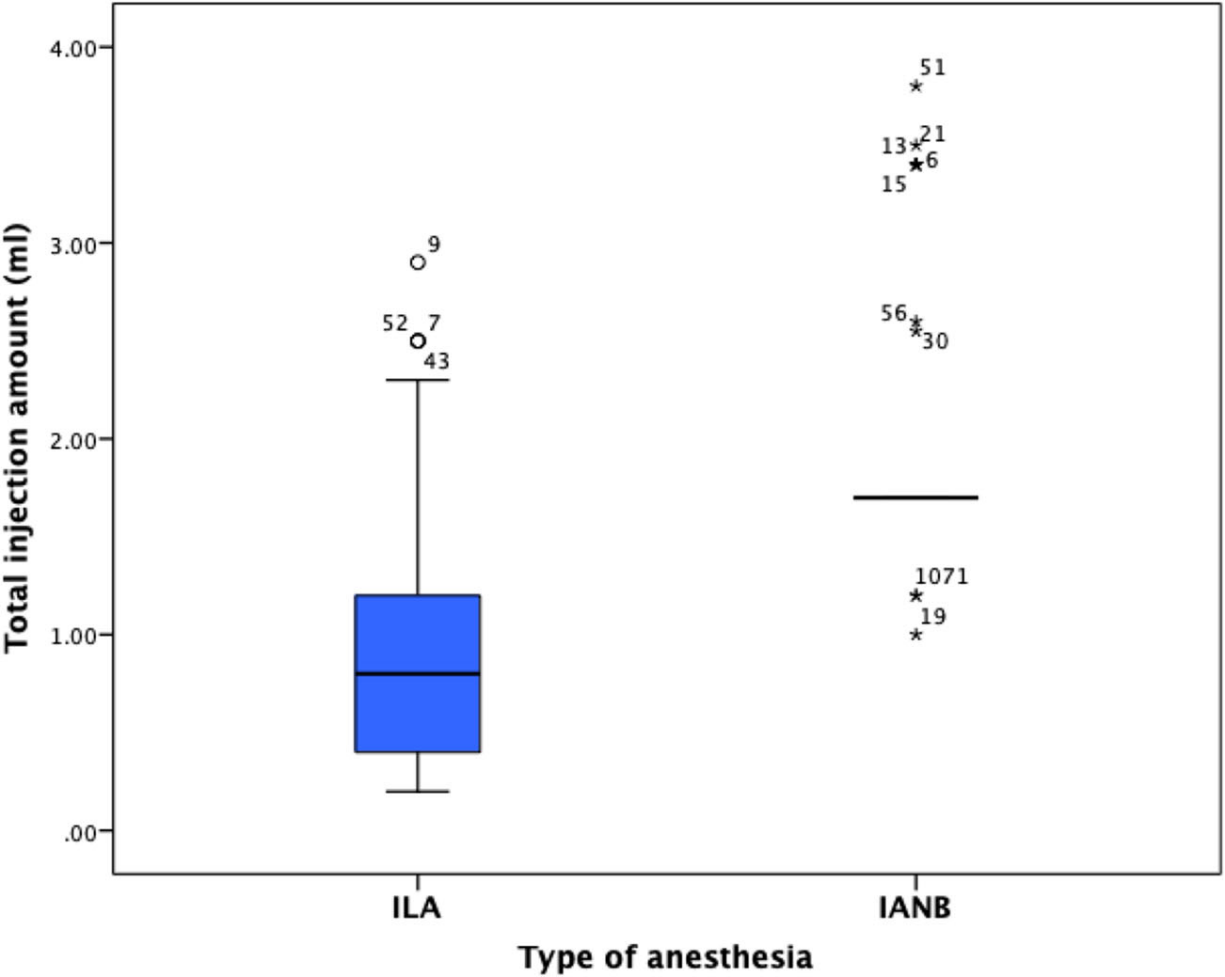
The total injection amount (ml) of local anesthetic solution during the entire procedure for inferior alveolar nerve block (IANB) vs. intraligamentary anesthesia (ILA)

Seventy-two adult patients (39 males, 33 females) were enrolled and participated in this study. They were randomizer located in two groups, IANB group (37 teeth were treated in 37 patients) and ILA group (35 teeth were treated in 35 patients). Both groups showed similar distributions of patients’ age, body mass, gender, treated teeth, and experience level of clinical instructors (Table 1). The performed treatments for IANB group were endodontic treatment (26%), caries removal (23%), and preparation of crown or inlay (23%, Table 2). For ILA group, they were replacement of old fillings (26%) and caries removal (23%, Table 2). The amount of local anesthesia that had been applied was significantly lower for ILA (*p* = 0.00, Fig. 2).

**Table 1:** Distribution of patients’ age, body mass, gender, treated teeth, and different experience level for clinical instructors and dental students in the 4th and 5th year for inferior alveolar nerve block (IANB) vs. intraligamentary anesthesia (ILA)

	IANB group	ILA group	*p* value
Age (years), mean (SD)	41.2 (12.1)	44.4 (16.4)	0.3
BMI (Kg/m^2^), mean (SD)	25.9 (4.4)	26.1 (5.9)	0.8
Gender, pat. no. (%)			0.3
Male	22 (59.5%)	17 (48.6%)	
Female	15 (40.5%)	18 (51.4%)	
Treated teeth. no. (%)			0.47
Molars	29 (78.3%)	26 (74.2%)	
Premolars	8 (21.6%)	9 (25.7%)	
Experience level no. (%).			0.9
Clinical instructor (GP)	8 (21.6%)	8 (22.8%)	
Dental students in the 5th year	20 (54.0%)	18 (51.4%)	
Dental students in the 4th year	9 (24.3%)	9 (25.7%)	

*SD*, standard deviation; *GP*, general practitioner

**Table 2:** Distributions of clinical treatment of permanent mandibular posterior teeth for inferior alveolar nerve block (IANB) vs. intraligamentary anesthesia (ILA)

Type of clinical treatment	IANB group	ILA group
Caries removal (%)	23%	23%
Endodontic treatment (%)	26%	18%
Preparation of crown or inlay (%)	23%	15%
Insertion of crown or inlay (%)	8%	12%
Replacement of old filling (%)	17%	26%
Extraction of single tooth (%)	3%	6%

### Pain of injection and during treatment

**Fig. 3:**
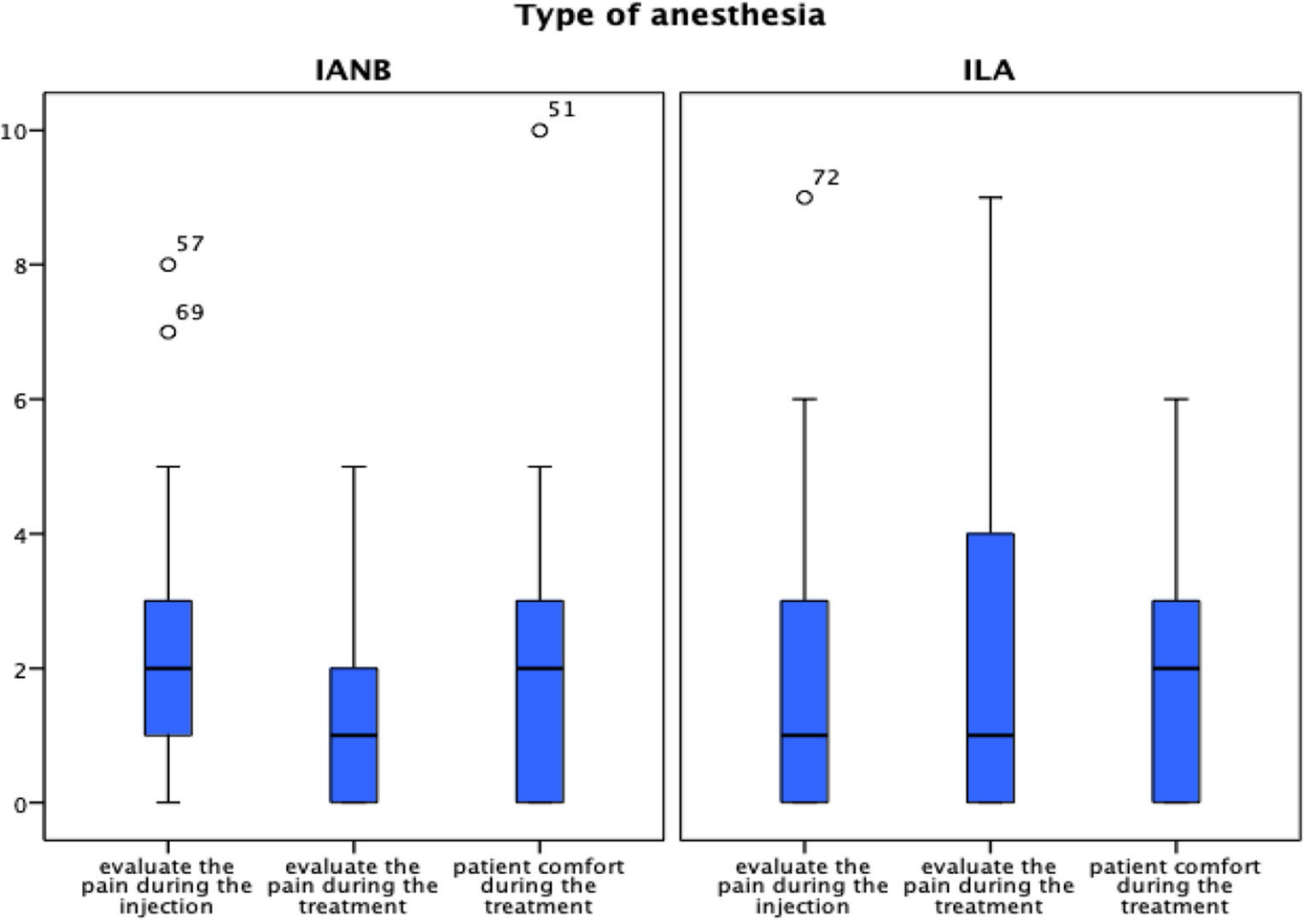
Assessment of the differences between pain of injection, pain during treatment, and unpleasantness of treatment for inferior alveolar nerve block (IANB) vs. intraligamentary anesthesia (ILA) by using a 10-point segmented numeric rating scale (NRS)

The pain of injection and during treatment assessed on the numeric rating scale (NRS) showed lower pain scores during the injection and treatment reported by the patients in ILA and IANB groups (Table 3). However, ILA showed significantly less pain during the injection than IANB (*p* = 0.03), while both techniques were similarly effective in pain control during the dental treatment (*p* = 0.2). In cases of ILA, 6 (17%) patients reported high scores of pain (> 5) during treatment and 2 (5.7%) patients during the injection; for IANB, 2 (5.4%) patients reported high scores of pain (> 5) during the injection (Fig. 3).

**Table 3:** The median pain score during injection, treatment, and patient’s comfort for inferior alveolar nerve block (IANB) vs. intraligamentary anesthesia (ILA) by using a 10-point segmented numeric rating scale (NRS)

Pain score	IANB group	ILA group	*p* value
Pain score during injection (0–10); median (IQR)	2 (2)	1 (3)	0.03
Pain score during treatment (0–10); median (IQR)	1 (2)	1 (5)	0.2
Patient comfort during the treatment (0–10); median (IQR)	2 (3)	2 (3)	0.7

*IQR*, interquartile range

The patient’s discomfort during treatment was very low and similar in both groups indicating mostly satisfactory local anesthesia (*p* = 0.7), but the distribution showed considerable variation within patients of both groups. One patient in each group rated the experience as unpleasant (> 5, Fig. 3).

### Frequency of complications or irritations

**Fig. 4:**
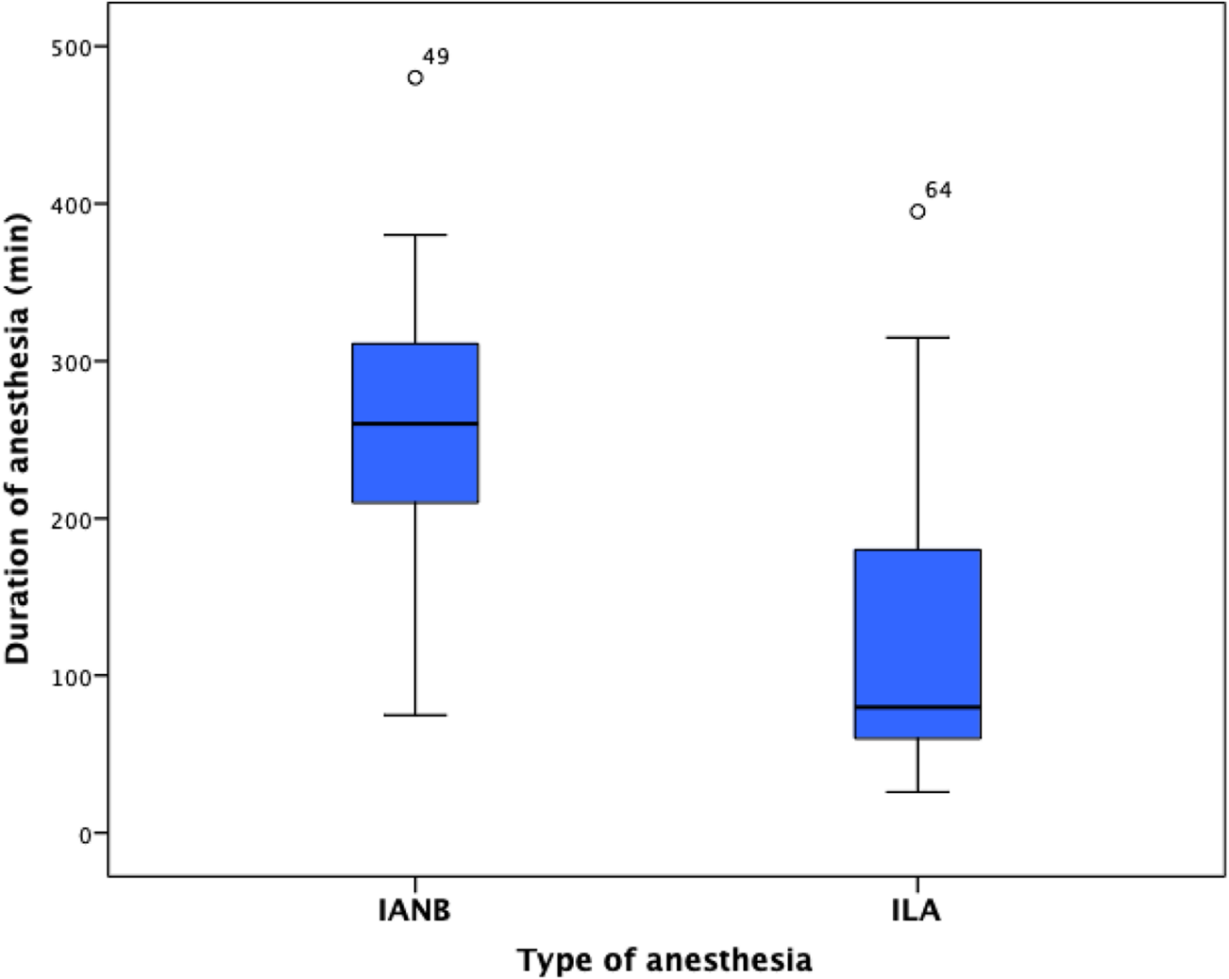
The duration of soft tissue anesthesia (min) for inferior alveolar nerve block (IANB) vs. for intraligamentary anesthesia (ILA)

In general, IANB had a longer time of local anesthesia than ILA (*p* = 0.00, Fig. 4). No sign of detrimental nerve contact or other complications were observed in any patient. However, one case with IANB reported difficulty during talking for 1 day after the anesthesia, other three more cases reported pain at the site of injection, and one case reported pain around the ear after the injection. On the other hand, no signs of any complication including soft tissue necrosis were observed with ILA technique.

### Comparison of different ILA syringes

**Fig. 5:**
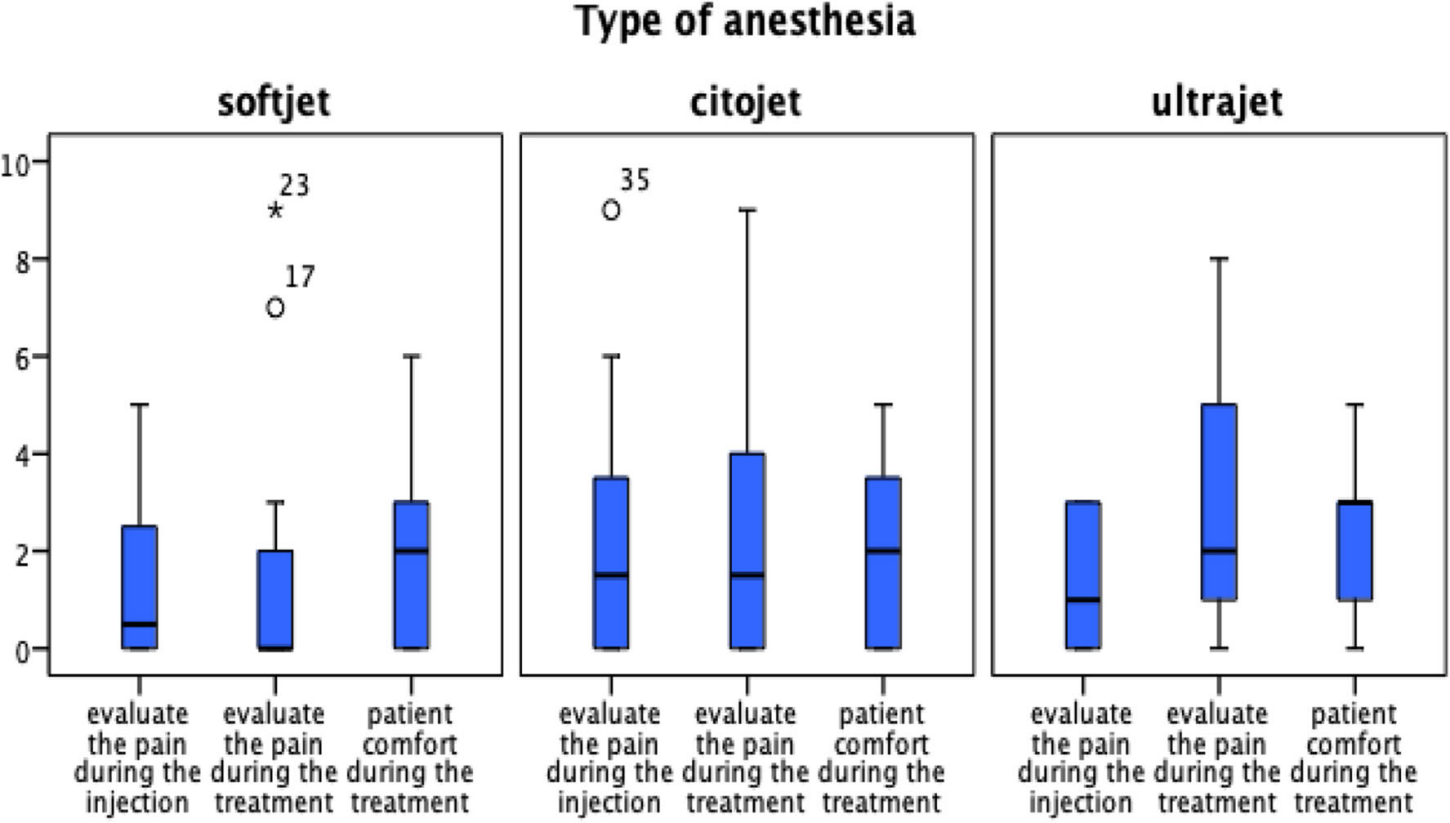
Assessment of the differences between pain of injection, pain during treatment, and unpleasantness of treatment for different intraligamentary syringe systems (Softjet, Citojet, and Ultrajet) by using a 10-point segmented numeric rating scale (NRS)

In two cases using Softjet syringes (16.6%) and Ultrajet (18.1%), high scores of pains (> 5) during following dental treatment were reported. For Citojet syringes, two patients (18%) reported high score of pain (> 5) during the injection and the subsequent treatment were reported. Still, the comparison of the pain parameters for the three different ILA syringes showed a very similar outcome (Table 4, Fig. 5). The comparison of the patient’s comfort during treatment resulted in an equivalent outcome with overall low degrees of unpleasantness (*p* = 0.7; Table 4). Only one patient rated the experience as unpleasant (> 5) for Softjet syringes (Fig. 5).

**Table 4:** Median pain score during injection, treatment, and patient comfort for different intraligamentary syringe systems (Softjet, Citojet, and Ultrajet) by using a 10-point segmented numeric rating scale (NRS)

Pain score	Softjet	Citojet	Ultrajet	*p* value
Pain score during injection (0–10); median (IQR)	0.5 (3)	1.5 (4)	1 (3)	0.6
Pain score during treatment (0–10); median (IQR)	0 (2)	1.5 (4)	2 (4)	0.3
Patient comfort during the treatment (0–10); median (IQR)	3 (2)	3 (3)	3 (2)	0.7

*IQR*, interquartile range

## Discussion

The results of this study indicate that ILA is at least as efficient as IANB in terms of effective pain control and the degree of unpleasantness for routine dental treatment as proposed by other studies and meta-analysis [5, 10, 11]. Similar to our study, Kämmerer et al. [10] reported an injection pain score of 1.55 ± 1.18 (mean ± SD) for mechanical (PDL-S) application and a 1.85 ± 1.22 (mean ± SD) computer-controlled (CCLAD) in 22 cases of ILA vs. 3.05 ± 1.99 (mean ± SD) in 20 cases of IANB (*p* = 0.005). Three years later, the same authors in another study confirmed the superiority of ILA and found a mean injection pain score of 2.19 ± 1.8 (mean ± SD) for ILA vs. 3.65 ± 1.9 (mean ± SD) for IANB (*p* < 0.00) [10, 11].

Although our study as well as other previously mentioned studies had reported a clear superiority of ILA over IANB for injection pain control, “regardless of the type of syringe systems used”, both anesthetic techniques showed no statistically significant differences concerning the success rate, the depth of the block, and pain scores during the subsequent dental treatment.

As the pain recognized by the patient is the most important reason for anxiety or phobia of dental treatment and the main aspect in the evaluation of the effectiveness of local anesthetic techniques [10, 11], thus pain assessment is crucial during dental treatment and the most commonly used tool is the numeric rating scale (NRS) from 0 to 10 as it is a highly reliable and appropriate for dental environment [18].

On contrast to our result, Dumbrigue et al. reported that the injections of ILA were associated with more pain and discomfort to the patient compared with IANB; however, the authors did not consider each extracted tooth as an independent sample [18]. Several intraligamentary injections were required in the same quadrant, which may be a reason for the great patients’ discomfort during the injections with ILA. Moreover, Dumbrigue et al. used pistol type syringes without safety pressure limiting mechanism, and the injection takes place rapidly under higher pressure than appropriate which increase the injection pain certainly [12]. Thus, the results of such studies with small sample size (16 patients, 45 teeth) should be taken cautiously. It must also be emphasized that the comparisons between different studies are difficult due to variable procedural factors possibly associated with pain experience during the administration of local anesthetic such as, the type or the amount of local anesthetic solution, temperature of the injection solution, injection rate, site of injection, and the experience of the dentist.

In addition, our study is in agreement with Kämmerer et al.’s [10] study which found no statistically significant difference in both groups regarding patient satisfaction (comfort) and over all pain experienced during the entire treatment course.

Our study confirmed ILA as a reliable alternative technique to IANB with the superiority of the following: the amount of local anesthesia for ILA was small and carries no risk of systemic toxicity with accidental intravascular injection [9, 15]. As ILA mostly wearing off at the end of the dental treatment with no residual regional anesthesia (as in case of LANB), due to the sensitivity of the lower lip for considerable time after the dental treatment, this reduces the risk of unwanted side effects such as lip biting in children or patients with mental disabilities who have higher risk of bite or thermal injury as reported in our and other previous studies [9, 15]; in addition, it also reduces the risk of temporary unpleasant reductions of mastication and speech.

## Conclusion

ILA has shown to be a safe and reliable method of local anesthesia for treatment of lower premolars and molars, with a success rate comparable to IANB without complications and temporary irritations. Thus, ILA can be considered as an effective alternative to IANB for routine dental treatment to reduce known side effects of IANB.

## Supplementary information

ESM 1 (DOC 33 kb)

ESM 2 (DOC 217 kb)
