# Supplementary material for: RCT on the effectiveness of the intraligamentary anesthesia and inferior alveolar nerve block on pain during dental treatment
Source: Clin Oral Investig. 2021 Feb 1;25(8):4825–32. doi: 10.1007/s00784-021-03787-x (PMC8342397; doi:10.1007/s00784-021-03787-x)
Supplement: Supplementary file 1 — (DOC 33 kb) [file 784_2021_3787_MOESM1_ESM.doc]

**CONSORT 2010 Flow Diagram**

**Allocation**

**Analysis**

**Follow-Up**

**Enrollment**

Assessed for eligibility (n= 72)

Excluded (n=0)

♦  Not meeting inclusion criteria (n=0)

♦  Declined to participate (n=0)

♦  Other reasons (n= 0)

Analysed (n=37)
♦ Excluded from analysis (give reasons) (n=0)

Lost to follow-up (give reasons) (n=0)

Discontinued intervention (give reasons) (n=0)

Allocated to intervention:(n=37)

♦ Received allocated intervention (n=37)

♦ Did not receive allocated intervention (give reasons) (n=0)

Lost to follow-up (give reasons) (n=0)

Discontinued intervention (give reasons) (n=0)

Allocated to intervention (n=35)

♦ Received allocated intervention (n=35 )

♦ Did not receive allocated intervention (give reasons) (n=0 )

Analysed (n=35)
♦ Excluded from analysis (give reasons) (n=0)

Randomized (n=72)

Experimental (n=35)

Control (n=37)
